# Translational Challenges and Strategies for Antibody Therapeutics in Glioblastoma

**DOI:** 10.3390/curroncol33100619

**Published:** 2026-10-14

**Authors:** Yangyi Zhou, You Li, Yu Chen, Peiqi Zhao, Bowen Wang, Haoliang Zhu, Hangzhe Sun, Tianyun Zhang, Anke Zhang, Yuanbo Pan, Kankai Wang

**Affiliations:** 1 The Second Affiliated Hospital, School of Medicine, Zhejiang University, Hangzhou 310009, China; yangyizhou@wmu.edu.cn (Y.Z.); zhaopeiqi1007@163.com (P.Z.); encore@zju.edu.cn (A.Z.); 2 The First Clinical Medical College, Wenzhou Medical University, Wenzhou 325000, China; liyou0506@wmu.edu.cn (Y.L.); haoliangzhu@wmu.edu.cn (H.Z.); hangzhesun@wmu.edu.cn (H.S.); tianyunzhang@wmu.edu.cn (T.Z.); 3 National Clinical Research Center for Ocular Diseases, Eye Hospital, Wenzhou Medical University, Wenzhou 325000, China; yuchen71@wmu.com; 4 Department of Neurosurgery, The First Affiliated Hospital of Bengbu Medical University, Bengbu 233000, China; 19872039462@163.com; 5 Department of Neurosurgery, The Second Affiliated Hospital, School of Medicine, Zhejiang University, Hangzhou 310009, China; 6 Zhejiang Key Laboratory of Research and Transformation for Major Neurosurgical Diseases, Hangzhou 310009, China

**Keywords:** glioblastoma, monoclonal antibodies, blood–brain barrier, tumor heterogeneity, tumor microenvironment

## Abstract

Glioblastoma (GBM) remains difficult to control. Monoclonal antibodies (mAbs) have transformed the treatment of several extracranial cancers, but they have not consistently improved survival in GBM. This narrative review examines the main biological and translational barriers to antibody-based therapy. It also evaluates strategies to overcome them. We reviewed preclinical, translational, and clinical evidence on antibody delivery, target stability, the tumor microenvironment, target discovery, and emerging antibody formats. Heterogeneous blood–brain and blood–tumor barriers produce uneven intratumoral exposure, especially at infiltrative margins. Spatial and temporal heterogeneity promotes antigen loss and treatment escape. Lymphocyte dysfunction, myeloid dominance, and suppressive vascular and metabolic programs further limit immune activity. Receptor-mediated transcytosis and focused ultrasound may improve delivery. Single-cell and spatial profiling, surfaceome and endocytome proteomics, longitudinal sampling, and causal inference may improve target selection. Depending on their design, antibody–drug conjugates (ADCs) can deliver cytotoxic payloads, whereas bispecific constructs can enable immune redirection, dual-target recognition, or receptor-mediated transport. However, clinical benefit remains limited by construct design, target accessibility, regional exposure, toxicity, and inadequate biomarker selection. Successful translation requires modality-specific alignment of exposure at the relevant site of action, target requirements, immune dependence, molecular design, and safety. Biomarker-guided patient selection, spatial pharmacokinetic and pharmacodynamic validation, and early evidence of clinical benefit are also essential.

## Simple Summary

Antibody treatments have improved care for several cancers, but their benefit in glioblastoma remains limited. Getting these drugs into the brain is part of the challenge: the tumor core may receive an antibody while infiltrating cells beyond it receive much less. Even when a tumor-directed antibody arrives, some cells may lack its target or lose it as the disease changes. Treatments that rely on T cells face an additional obstacle in the suppressive immune environment. This review examines how these problems affect different antibody therapies and discusses ways to improve delivery, choose more reliable targets, and design new treatments. Early studies should measure drug exposure and biological effects in different tumor regions, alongside safety and patient outcomes. These measurements may help explain why a promising treatment falls short and guide decisions about larger trials.

## 1. Introduction

Glioblastoma (GBM) remains difficult to control because treatment must address disease beyond the contrast-enhancing tumor. Under the 2021 World Health Organization classification of central nervous system tumors (WHO CNS5), GBM is an IDH-wildtype entity (CNS WHO grade 4); tumors previously termed IDH-mutant GBM are now classified as astrocytoma, IDH-mutant, CNS WHO grade 4 [1]. Some historical GBM trials and molecular cohorts therefore may not correspond exactly to the current WHO-defined entity, particularly when IDH status was not reported. The biological challenge extends across several compartments: infiltrative tumor regions can be difficult to expose to systemically administered antibody constructs [2], target expression can change between primary and recurrent disease [3], and the immune microenvironment can constrain antitumor effector function [4]. These features complicate the translation of a positive tumor biomarker into durable whole-tumor control.

Monoclonal antibodies (mAbs) nevertheless offer a strong therapeutic rationale. Trastuzumab established the value of biomarker-guided HER2 targeting in metastatic breast cancer [5]. Bevacizumab improved survival when added to chemotherapy for metastatic colorectal cancer [6], while pembrolizumab produced durable benefit in PD-L1-high metastatic non-small-cell lung cancer [7]. Antigen-specific recognition can widen the therapeutic window; the fragment crystallizable (Fc) region can recruit immune effectors; and recycling through the neonatal Fc receptor (FcRn) can prolong systemic exposure. Humanization and molecular engineering have also expanded the range of antibody formats and enabled repeated dosing [8,9]. In GBM, however, these advantages matter only when the construct reaches its required site of action, encounters a sufficiently stable target, and operates in a biological context compatible with its mechanism.

Clinical experience illustrates the gap between these requirements and observed benefit. In the phase III AVAglio trial in newly diagnosed GBM, adding bevacizumab to radiotherapy and temozolomide improved progression-free survival but not overall survival [10]. In RTOG 0825, progression-free survival was numerically longer but did not meet the prespecified significance threshold, and overall survival was not improved [11]. VEGF blockade can reduce vascular permeability, edema, and contrast enhancement, so an apparent radiographic response may coexist with persistent non-enhancing infiltrative disease [12,13]. Phase III nivolumab trials also failed to improve survival: CheckMate 498 tested nivolumab in place of temozolomide in MGMT-promoter-unmethylated disease, whereas CheckMate 548 added it to chemoradiotherapy in MGMT-promoter-methylated disease [14,15]. Depatuxizumab mafodotin likewise did not improve overall survival despite selection for EGFR-amplified tumors [16]. These outcomes do not establish a single shared cause of failure. Antiangiogenic antibodies, checkpoint inhibitors, and tumor-targeting antibody–drug conjugates differ in their required sites of action and dependence on tumor antigens or immune effectors.

A useful translational framework must therefore match each antibody format to its particular requirements for regional exposure, target accessibility and stability, immune activity, and safety. This Review first examines heterogeneous delivery across the blood–brain and blood–tumor barriers, spatiotemporal target instability, and immune suppression. It then evaluates strategies for delivery and target discovery, experimental validation, and emerging antibody formats. Throughout, we emphasize spatial pharmacokinetic and pharmacodynamic assessment, treatment-state-matched biomarkers, and early clinical studies that can distinguish inadequate delivery from target or mechanism failure. These steps may help connect target engagement to durable clinical benefit in GBM.

## 2. Barriers to Monoclonal Antibody Efficacy in Glioblastoma

**Figure 1:**
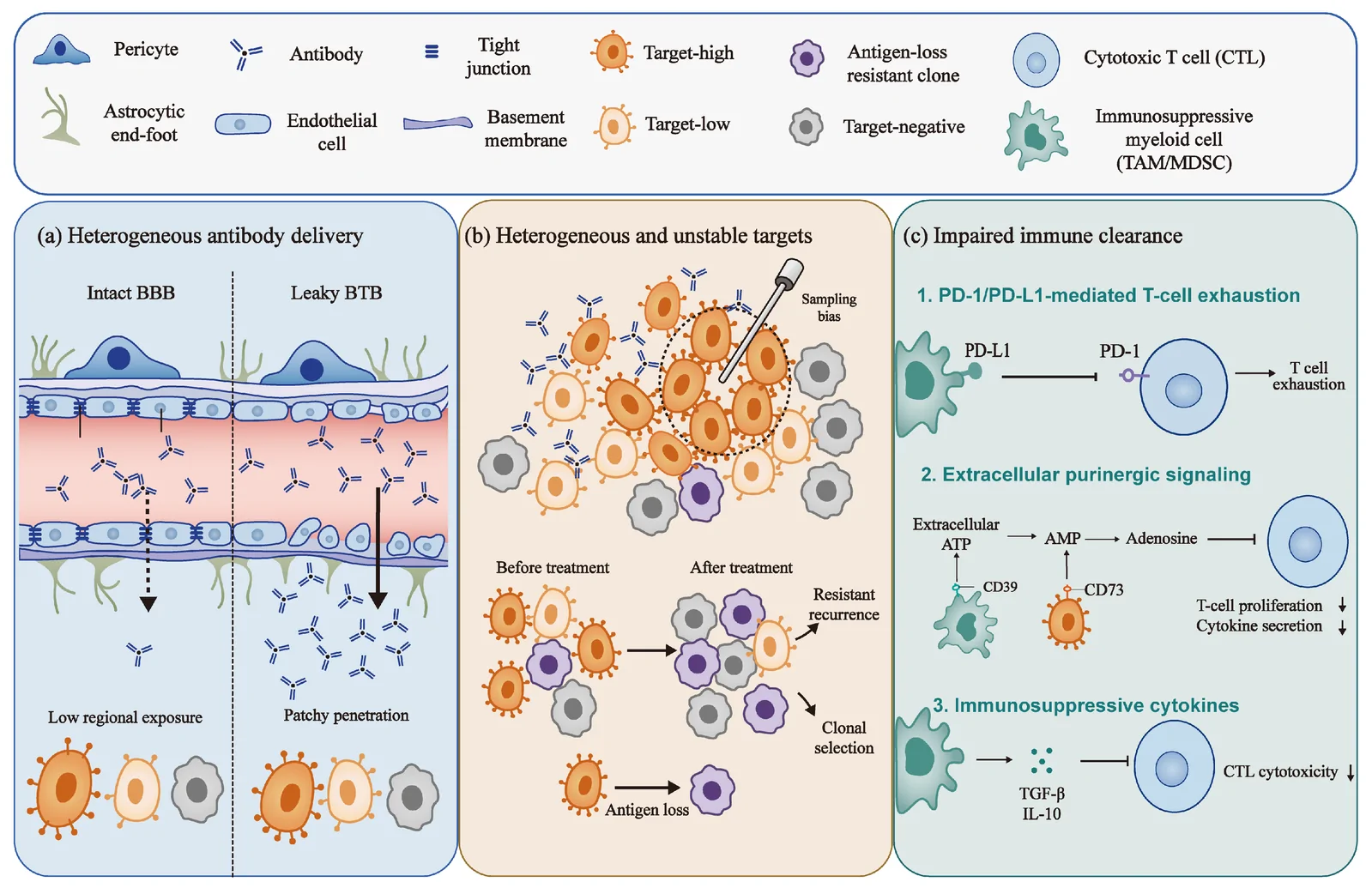
Major barriers to monoclonal antibody efficacy in glioblastoma. (**a**) The intact blood–brain barrier (BBB) and heterogeneously leaky blood–tumor barrier (BTB) produce low or patchy regional antibody exposure. The dashed arrow indicates restricted antibody entry across the intact BBB, whereas the solid arrow indicates relatively greater antibody entry through leaky regions of the BTB. (**b**) Target heterogeneity and treatment-associated antigen loss promote clonal selection and resistant recurrence. (**c**) The schematic depicts PD-L1 on a suppressive myeloid cell engaging PD-1 on a cytotoxic T cell, CD39/CD73-mediated conversion of extracellular ATP to immunosuppressive adenosine, and myeloid-derived TGF-β and IL-10 restraining T-cell activity. The illustrated cellular sources and marker patterns may vary among tumors. Abbreviations: BBB, blood–brain barrier; BTB, blood–tumor barrier; CTL, cytotoxic T lymphocyte; IL-10, interleukin-10; MDSC, myeloid-derived suppressor cell; PD-1, programmed cell death protein 1; PD-L1, programmed death ligand 1; TAM, tumor-associated macrophage; TGF-β, transforming growth factor β.

Three interrelated barriers have limited the clinical translation of monoclonal antibodies and antibody-based constructs in glioblastoma (GBM). These barriers are heterogeneous delivery across the blood–brain barrier (BBB) and blood–tumor barrier (BTB), spatiotemporal instability of target expression, and an immunosuppressive microenvironment that restricts antibody effector function. These three interrelated barriers are summarized in Figure 1.

### 2.1. Heterogeneous Antibody Delivery Across the BBB and BTB

An antibody given intravenously has limited access to brain tissue where the BBB remains intact. Brain endothelial cells form tight junctions and have low rates of nonspecific vesicular transport. Pericytes, the basement membrane, and astrocytic end-feet help maintain this barrier. A tumor cell may therefore express the intended target but still receive too little antibody for treatment to work. Together, these features restrict paracellular entry and passive transcellular diffusion [17,18,19]. A full-length immunoglobulin G (IgG) or antibody–drug conjugate (ADC) is approximately 150 kDa or larger and therefore lacks an efficient passive route into the brain parenchyma. Entry generally requires receptor-mediated transcytosis, deliberate barrier disruption, or direct bypass of the barrier [20,21,22]. Brain penetration of conventional antibodies is often reported as less than 0.1%. However, this value should be treated as an order-of-magnitude indication of very low exposure rather than as a universal biodistribution constant. Brain-to-plasma, cerebrospinal-fluid-to-plasma, and percentage-of-injected-dose-per-gram measurements represent different compartments and are not interchangeable [17,23]. The relevant pharmacological question is not whether an antibody can be detected in the brain. Instead, it is whether pharmacologically active exposure exceeds the required threshold throughout the tumor.

GBM does not uniformly abolish the BBB. Instead, it creates a spatially heterogeneous blood–tumor barrier (BTB). Abnormal vessels and VEGF-associated permeability permit macromolecular leakage in parts of the contrast-enhancing core. By contrast, non-enhancing invasive margins and some regions within enhancing lesions may retain restrictive endothelium. Imaging and mechanistic studies show that permeability, perfusion, transcytosis, and efflux vary both between and within lesions. Clinical and imaging evidence indicates that infiltrative GBM can extend into regions with a relatively intact BBB. Amino-acid positron emission tomography (PET)-defined disease frequently extends beyond the contrast-enhancing volume [18,19,20,21]. Human tissue studies support this spatial mismatch. After preoperative intravenous administration of the murine anti-EGFR antibody EMD55900, only approximately 0.0001–0.00001% of the injected dose per gram was recovered from resected brain tumor at 48 h. This low recovery occurred despite dose-dependent EGFR occupancy in the sampled tissue [23]. In a separate first-in-human imaging study, cetuximab-IRDye800 fluorescence was higher in two contrast-enhancing GBMs than in one non-enhancing grade II diffuse astrocytoma. The non-enhancing astrocytoma showed EGFR positivity on immunohistochemistry but no detectable fluorescence; because this case was not GBM, the comparison does not directly establish antibody exposure at the infiltrative margin of GBM [24]. Thus, target engagement in a surgically accessible region cannot be extrapolated to uniform coverage of diffusely infiltrating disease. This heterogeneous access is illustrated in Figure 1a.

Even after an antibody crosses a tumor vessel, the extracellular matrix (ECM) may limit its movement through tumor tissue and contribute to uneven regional exposure. Hyaluronan-rich extracellular matrices can influence invasion by patient-derived GBM cells in engineered three-dimensional systems [25]. In experiments using subcutaneous tumor xenografts, including U87 glioma, collagen network organization was associated with resistance to interstitial IgG diffusion; collagenase increased IgG mobility in the more resistant tumors [26]. These findings support a plausible postvascular source of uneven antibody exposure, although its magnitude in patients with GBM and the clinical value of ECM remodeling remain to be established.

Bevacizumab illustrates why vascular and imaging responses cannot be equated with whole-tumor exposure. In RTOG 0825, progression-free survival was numerically longer but did not meet the prespecified significance threshold, and overall survival was not improved [11]. Reduced permeability and edema do not, by themselves, demonstrate effective exposure of infiltrative disease [11,20,27]. This clinical pattern primarily reflects vascular control and cannot be attributed simply to an inability to cross the BBB.

Depatux-M provides a more direct example of a delivery limitation. INTELLANCE-1 found no overall survival benefit and only a limited progression-free survival signal in newly diagnosed, EGFR-amplified GBM [16,28]. However, the trial could not attribute treatment failure solely to the BBB. In patient-derived xenograft mouse models, most EGFR-amplified tumors were sensitive at heterotopic sites, whereas only two of seven responded intracranially. Poor orthotopic activity was associated with limited and heterogeneous ADC distribution. Activity improved after BBB disruption or intracranial delivery [2]. Regional exposure was therefore one limiting factor, together with target heterogeneity and construct pharmacology.

Plasma pharmacokinetics, contrast enhancement, and a positive measurement from a single resection specimen are therefore inadequate surrogates for whole-tumor exposure. Phase 0 tumor sampling has directly quantified intratumoral payload exposure after sacituzumab govitecan, whereas early pulsed-ultrasound and superselective intra-arterial studies have primarily established the feasibility and safety of transient barrier disruption or regional administration. Collectively, these studies have not demonstrated spatially uniform antibody coverage or a survival benefit [18,22,29,30,31]. The central problem is heterogeneous exposure rather than a complete absence of exposure. Improving delivery is necessary but insufficient. Adequate exposure must coincide with a target that remains abundant and stable across space and time.

### 2.2. Spatiotemporal Tumor Heterogeneity and Therapeutic Target Instability

GBM varies from one region to another even before treatment. Image-localized biopsies have found different EGFR, PDGFRA, and PTEN alterations within the same tumor [32]. Spatial analysis of human GBM biopsies has also identified rare THY1-positive, mesenchymal-like regions near blood vessels [33]. A surgical specimen describes the region sampled and may miss cells at the invasive edge or elsewhere. For a tumor-directed antibody, high average antigen expression in one specimen does not mean that every malignant cell carries the target. This heterogeneity suggests a possible route to escape: target-low or target-negative cells could remain after target-positive cells are eliminated.

The tumor may change further by the time it recurs. Comparisons of diagnostic and recurrent samples suggest that some relapse-associated clones diverged before diagnosis and that treatment favored some branches over others [34,35]. EGFR, PTEN, and NF1 alterations may be present at only one of the two time points, while some recurrent tumors acquire mismatch-repair defects or a temozolomide (TMZ)-associated hypermutator phenotype [34,35,36]. This does not mean that TMZ invariably produces antigen-negative cells. In a patient-derived orthotopic mouse model, rare THY1-positive mesenchymal/stem-like cells present before treatment preferentially repopulated tumors after TMZ and irradiation [33]. Experiments have also linked TMZ tolerance and hypermutation to interactions between mismatch repair and RAD18-dependent translesion synthesis [37]. Recurrence can thus reflect expansion of pre-existing cells, treatment-associated mutations, and shifts in cell state. Figure 1b illustrates how a single target may become less representative of the tumor over time.

EGFR illustrates why biomarkers within the same pathway may differ in longitudinal stability. In paired samples obtained after uniform chemoradiation, EGFR amplification was retained in most recurrent tumors. By contrast, approximately half of tumors that were initially positive for epidermal growth factor receptor variant III (EGFRvIII) no longer expressed EGFRvIII at recurrence [3]. This difference is important because EGFR amplification, EGFRvIII expression, and surface accessibility are related but non-equivalent properties. A trial that enrolls a patient at recurrence on the basis of an archival diagnostic specimen may select for an alteration that persists at the DNA level while overestimating the abundance or accessibility of the recognized epitope. The preservation of a driver alteration also does not prove continued functional dependence. Integrated proteogenomic profiling has shown that recurrent GBM can adopt neuronal-like and synaptogenic states even when several canonical genomic drivers remain conserved at the cohort level [36,38]. The phase III ACT IV trial provides an instructive clinical example of this temporal mismatch. Rindopepimut is an EGFRvIII-directed peptide vaccine rather than a monoclonal antibody, but the trial tested whether a single tumor-specific antigen identified at diagnosis could sustain selective therapy. In patients with centrally confirmed EGFRvIII-positive newly diagnosed GBM, rindopepimut plus maintenance TMZ induced a robust humoral response but did not improve overall survival compared with control treatment. EGFRvIII was absent in approximately 60% of evaluable recurrent specimens, and loss occurred frequently in both treatment groups [39]. Because EGFRvIII loss was frequent in both treatment groups, ACT IV does not show that rindopepimut caused antigen escape. The findings are consistent with pre-existing EGFRvIII heterogeneity and target instability, but the trial did not establish how much these mechanisms, or immune suppression, contributed to the absence of a survival benefit. ACT IV therefore provides a warning for antibody development. An antigen-specific immune response does not ensure elimination of antigen-negative or antigen-losing tumor cells. The immunosuppressive GBM microenvironment and limited availability of functional effector cells may also have weakened the translation of an EGFRvIII-specific immune response into tumor clearance, although ACT IV did not establish the contribution of either mechanism.

Depatux-M extends this problem to an ADC. Early studies in recurrent GBM showed activity, but EGFRvIII did not consistently predict progression-free or overall survival [40,41]. INTELLANCE-1 similarly showed a limited progression-free survival signal without an overall survival benefit in EGFR-amplified disease [16]. Depletion of EGFR-amplified or EGFRvIII-positive cells followed by resistant outgrowth is compatible with target-mediated selection, but this mechanism was not established as the sole explanation. Delivery heterogeneity, biomarker enrichment, payload pharmacology, and ocular toxicity remain alternative or complementary explanations. Baseline EGFR amplification alone therefore did not identify a population with uniform exposure, persistent antigen expression, and durable target dependence.

Target escape may occur without deletion of the target gene or complete loss of its protein. Paired single-cell analyses from a nivolumab-treated GBM cohort associated immunotherapy with increased mesenchymal stem-like tumor states, tumor-associated macrophages, and exhausted or proliferative T-cell populations [42]. Together with longitudinal proteogenomic evidence of recurrence-associated state transitions, these findings broaden the concept of target loss to include functional escape. The antigen may remain detectable, but the cellular program, signaling dependency, or microenvironment required for antibody activity may have changed [38,42]. This distinction avoids a common interpretive error in negative trials: equating biomarker positivity with sustained therapeutic susceptibility.

Taken together, GBM heterogeneity turns single-target therapy into an evolutionary filter. Target assessment should reflect the tumor state at treatment initiation. Where feasible, it should use recurrent-tumor, multiregional, or longitudinal measurements rather than an archival single-site result. Multi-antigen strategies may broaden coverage, but antigen loss must still be distinguished from inadequate exposure. Tumor evolution also remodels immune and myeloid compartments. Thus, even a retained target may be ineffective when the immune context cannot support antibody effector function. Representative human evidence is summarized in Table 1.

### 2.3. Immunosuppressive Microenvironment and Impaired Antibody Function

Even when antibodies reach their relevant sites of action, immune-dependent therapies may fail if the required effector cells are absent or dysfunctional. Checkpoint inhibitors act on immune signaling, whereas Fc-dependent tumor-targeting antibodies require effective interactions between antibody-coated tumor cells and immune effectors. T cells are often sparse in these tumors, and those that enter may have limited activity. Macrophages and other myeloid cells make up much of the immune infiltrate and can support immune suppression, vessel growth, and tissue repair. Hypoxia, inhibitory cytokines, suppressive metabolites, and treatment-related effects can further weaken antitumor responses [48,49,50]. Better antibody delivery may increase target binding, but it cannot by itself restore the immune activity needed to clear the tumor.

The shortage of therapeutically competent T cells begins outside the tumor. Patients with GBM may have profound lymphopenia before treatment. Translational studies further suggest that many naive T cells are sequestered in the bone marrow in association with loss of surface sphingosine-1-phosphate receptor 1. Reversing this sequestration restored the activity of T-cell-directed therapy in intracranial mouse tumor models. These findings support the principle that checkpoint blockade may fail when the circulating effector-cell pool is depleted or inaccessible [51]. T cells that enter GBM face a second barrier. Patient-derived tumor-infiltrating lymphocytes coexpress programmed cell death protein 1 (PD-1), LAG-3, TIGIT, and CD39 and show transcriptional and functional features of severe exhaustion [52,53]. PD-1 expression alone should not be equated with reversible exhaustion or therapeutic susceptibility. The concurrence of multiple inhibitory receptors and reduced cytokine responsiveness indicates that blocking one checkpoint may leave parallel suppressive programs intact.

This limited and dysfunctional lymphoid compartment is embedded in a heterogeneous but predominantly myeloid immune landscape. Analyses of human brain tumor specimens, complemented by cross-species single-cell studies, distinguish tissue-resident microglia from monocyte-derived macrophages and identify activation programs that cannot be represented by a binary M1/M2 classification [54,55]. Nevertheless, subsets with M2-like, lipid-associated, hypoxia-associated, or other immunosuppressive features can inhibit T-cell activity and promote tumor-supportive remodeling. This compartment also evolves with disease. Microglia-derived tumor-associated macrophages are prominent in newly diagnosed tumors, whereas monocyte-derived populations become more abundant in recurrent and hypoxic GBM. An antibody selected according to the immune context of the diagnostic specimen may therefore encounter a different set of Fc receptor-bearing effectors and suppressor cells at recurrence.

Interacting vascular, cytokine, and metabolic circuits sustain this suppressive state. TGF-β and IL-10 can limit cytotoxic lymphocyte activity, while CD39/CD73-dependent adenosine production, hypoxia, acidity, and VEGF favor regulatory T cells and suppressive myeloid states [49,50]. Abnormal blood vessels may contribute to this environment as well as affect drug entry. A study combining human GBM tissue, paired samples from bevacizumab-treated patients, and experimental models found an association between PLVAP-positive vessels and glioma-associated macrophage activation. It also identified α1-acid glycoprotein as a possible mediator of macrophage-supported tumor growth [27]. These observations suggest that vascular changes and myeloid suppression may reinforce one another. For a tumor-targeting IgG, however, the presence of Fcγ receptor-bearing myeloid cells does not establish that they will phagocytose antibody-coated tumor cells. Their functional state may instead favor tissue repair and immune suppression [54,55]. A recent study of plasma cells, IgG, and FcγRIIA also suggests possible unintended Fc-related signaling in glioma stem-like cells. However, this observation should not be generalized to all therapeutic antibodies or interpreted as established clinical harm [56]. These convergent suppressive circuits are depicted in Figure 1c.

Beyond tumor-associated macrophages and microglia, myeloid-derived suppressor cells (MDSCs) form an additional, functionally defined suppressive compartment. Blood samples from patients diagnosed with GBM showed increased MDSCs, and their ex vivo depletion partially restored T-cell interferon-γ production [57]. Single-cell and spatial analyses of IDH-wildtype GBM further identified distinct MDSC states, including a hypoxia-associated early progenitor population in pseudopalisading regions [58]. Regulatory T cells (Tregs) add a lymphoid constraint. In a mouse GBM model, an anti-CD25 antibody that preserved IL-2 signaling depleted Tregs, expanded CD8+ T-cell clones, increased FcγR-high macrophages, and enhanced responses to anti-PD-1 and EGFRvIII-targeting antibodies. Human GBM explants also showed immune activation, but benefit in patients has not been established [59]. These findings are most directly relevant to checkpoint blockade and Fc-dependent tumor-targeting antibodies.

GBM-derived extracellular vesicles (EVs) provide another route of intercellular communication. In cell-based experiments, GBM microvesicles transferred RNA and proteins to recipient cells and promoted endothelial tube formation and glioma-cell proliferation [60]. In separate co-culture experiments, PD-L1-bearing GBM EVs inhibited T-cell activation, and anti-PD-1 blockade reversed this effect when EV-associated PD-L1 was present [61]. Other GBM EV preparations instead generated suppressive monocytes, including MDSCs, while showing little direct T-cell inhibition [62]. Thus, EV-mediated immune effects depend on the experimental context; whether EVs predict resistance to checkpoint antibodies in patients remains unknown.

Randomized trials establish the limited clinical efficacy of the tested checkpoint-based regimens, but do not identify the mechanisms responsible. In CheckMate 498, nivolumab plus radiotherapy did not improve overall survival compared with temozolomide plus radiotherapy in newly diagnosed, MGMT-promoter-unmethylated GBM. Tumor PD-L1 expression did not identify a subgroup that benefited [14]. This result establishes that PD-1 blockade cannot replace standard therapy in the studied population. It does not identify poor brain penetration or any single immune mechanism as the cause. Preclinical and translational findings suggest that effector-cell scarcity, exhaustion, and myeloid suppression may contribute to resistance, but their relative contributions to these trial outcomes remain unresolved. A randomized phase II study in recurrent GBM similarly found limited activity with pembrolizumab alone or with bevacizumab, without a durable survival tail. PD-L1 expression, tumor-infiltrating lymphocyte density, and an immune gene-expression profile were not associated with survival [46]. VEGF inhibition may alter vascular permeability and parts of the myeloid niche. The limited activity of the tested combination is consistent with persistent immune barriers, but does not establish which suppressive mechanisms were responsible [46].

Evidence that individual components of the immune environment can be modified does not by itself establish clinical benefit. In a single-arm phase II study, the prespecified OS-12 threshold was met, and post-treatment tumors contained fewer MDSCs. However, without a randomized control, neither the survival effect nor its attribution to myeloid modulation can be established [47]. In a small randomized study of resectable recurrent GBM, neoadjuvant pembrolizumab induced intratumoral interferon-γ and T-cell programs, peripheral immune changes, and an exploratory survival signal compared with postoperative treatment alone [63]. A subsequent single-arm expansion cohort supported pharmacodynamic effects on tumor gene-expression programs but did not confirm a survival benefit [64]. These findings suggest that treatment timing may influence immune activation, but the survival benefit of neoadjuvant pembrolizumab remains unconfirmed. Overall, the GBM microenvironment disrupts antibody therapy downstream of delivery and target recognition. Effective strategies will probably need to recruit or prime effector cells, reverse exhaustion, and reprogram suppressive myeloid, vascular, and metabolic niches. Adding one checkpoint antibody to an otherwise unchanged immune ecosystem is unlikely to be sufficient. The convergence of heterogeneous delivery, unstable targets, and impaired immune clearance is summarized in Figure 1a–c.

## 3. Strategies for Antibody Delivery, Target Discovery and Validation

### 3.1. Receptor-Mediated and Emerging Strategies for Antibody Delivery

BBB integrity varies across the GBM landscape. A systemically administered antibody may therefore reach the contrast-enhancing core but remain subtherapeutic in infiltrative, non-enhancing disease. Advanced delivery strategies should be evaluated according to regional, pharmacologically active exposure rather than BBB opening or whole-brain uptake alone. Two leading approaches address different aspects of this problem. Receptor-mediated transcytosis (RMT) incorporates transport capacity into the molecule, whereas focused ultrasound with microbubbles (FUS/MB) creates a regional window for access.

**Figure 2:**
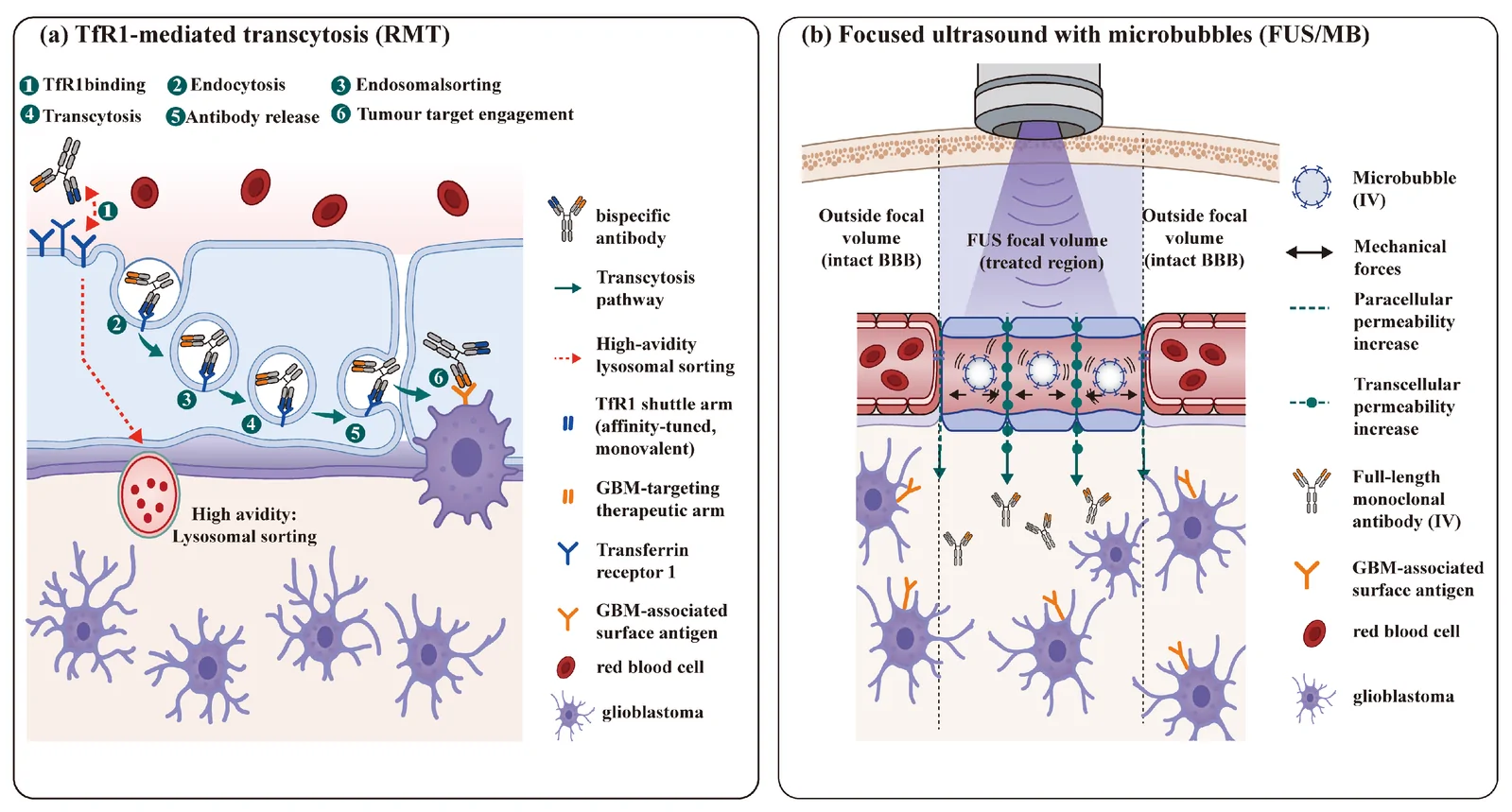
Strategies to enhance antibody delivery across the blood–brain barrier in glioblastoma. (**a**) Affinity-tuned, monovalent transferrin receptor 1 (TfR1)-mediated transcytosis enables endothelial transport and tumor engagement, whereas excessive avidity favors lysosomal sorting. (**b**) Focused ultrasound with microbubbles (FUS/MB) transiently increases focal paracellular and transcellular permeability; excessive cavitation may cause vascular injury. Abbreviations: BBB, blood–brain barrier; FUS/MB, focused ultrasound with microbubbles; TfR1, transferrin receptor 1.

RMT uses endogenous endothelial pathways, most commonly the transferrin receptor 1 (TfR1), to transport a therapeutic antibody or cargo across the BBB. In a mouse model of Alzheimer’s disease, a monovalent Brain Shuttle enhanced brain target engagement by an anti-amyloid-β antibody [65]. TfR/BACE1 bispecific antibodies with optimized TfR binding also demonstrated brain delivery in nonhuman primates [66]. The latter study revealed species-selective binding and an approximately fourfold difference in affinity between humans and monkeys [66]. Thus, affinity windows should not be transferred directly between species. High-affinity engagement may promote endothelial retention, TfR downregulation, and lysosomal sorting. Lower-affinity variants may improve brain exposure by reducing target-mediated clearance and receptor degradation [67]. Fc transport vehicles further support the feasibility of a modular, IgG-like shuttle architecture in mice and nonhuman primates [68]. Evidence specific to GBM remains preclinical. A pH-responsive anti-PEG × anti-TfR engager that dissociates in acidic endosomes enhanced delivery of PEGylated liposomal doxorubicin in an orthotopic human GBM xenograft mouse model [69]. A TfR-targeted aptamer–drug conjugate used endothelial and tumor-cell TfR as a dual-targeting route [70]. Neither study delivered a therapeutic monoclonal antibody as its cargo; thus, these results do not establish therapeutic antibody delivery or efficacy in GBM. Endothelial escape, coverage of infiltrative regions, peripheral TfR binding, and species-dependent pharmacology remain important translational constraints. The sequence from TfR1 binding and endocytosis to endosomal sorting, transcytosis, abluminal release, and GBM target engagement is illustrated in Figure 2a.

TfR1 and TfR2 should be distinguished when considering selective delivery. The antibody shuttle studies discussed above primarily target TfR1, which is also expressed by normal brain endothelium. These TfR1 shuttle studies support transport feasibility, but do not establish selective delivery to GBM-associated vasculature based on differential receptor expression relative to the normal BBB. In a human immunohistochemical study of 35 historically classified GBMs, TfR2 staining was detected in tumor-associated arteriolar and capillary endothelium in 21 cases, whereas normal brain vessels showed little or no staining [71]. This difference raises the possibility of tumor-vascular targeting, but expression alone does not establish luminal accessibility, antibody transcytosis, or delivery to infiltrative regions with a relatively intact BBB. Confirmation in contemporary IDH-wildtype GBM specimens and direct transport studies are needed before TfR2 can be considered a validated, tumor-selective BBB shuttle.

TfR1 is not the only receptor-mediated delivery route under investigation. CD98hc-targeting antibody transport vehicles increased brain exposure in humanized mice and nonhuman primates [72], while a separate bispecific-antibody study showed longer brain retention than a matched TfR1 shuttle in mice [73]. Angiopep-2 conjugation increased intracranial tumor accumulation of an anti-EGFR antibody in mice, but did not establish therapeutic efficacy, the specific contribution of LRP1-mediated transport, or coverage of infiltrative human GBM [74]. Related Angiopep-2-modified DNA nanostructures have coupled brain entry with tumor-context-dependent siRNA release: an NCL-responsive framework delivered siMGMT, whereas a later cascade additionally integrated acidic pH and glutathione to release siPARP1 in TMZ-resistant GBM models [75,76]. These non-antibody studies illustrate the value of separating transport from tumor-restricted payload activation, but do not establish therapeutic antibody delivery or efficacy in GBM. Receptor expression, endothelial trafficking, peripheral binding, and regional tumor exposure therefore require construct-specific assessment.

FUS/MB modifies the barrier rather than the payload. Acoustic activation of intravenously administered microbubbles transiently increases vascular permeability within a selected volume. This process creates a spatially and temporally controlled window for systemic drug delivery [77]. In a phase I study of recurrent GBM, paired biopsies of non-enhancing peritumoral brain showed a 3.7-fold increase in paclitaxel concentration after low-intensity pulsed ultrasound with microbubbles. An ancillary pharmacokinetic cohort from a related carboplatin trial showed a 5.9-fold increase in carboplatin concentration in sonicated versus non-sonicated peritumoral tissue [78,79]. A subsequent phase I/II SonoCloud-9 study in 33 patients expanded the opened volume and established the feasibility of repeated treatment. However, its single-arm design could not isolate a survival effect of BBB opening [79]. Noninvasive MR-guided FUS provides a complementary approach, and acoustic-emission feedback permits patient-specific power titration. In a phase 0 glioma study, effective power varied by almost an order of magnitude among patients. This variation argues against the use of a universal nominal setting [80]. Antibody-specific evidence should be interpreted within the same hierarchy. MR-guided FUS increased trastuzumab uptake in HER2-positive brain metastases, demonstrating macromolecular delivery in humans but not efficacy in GBM [81]. More directly, low-intensity pulsed ultrasound with microbubbles increased anti-PD-1 concentrations in glioma models and in peritumoral samples from a small cohort with recurrent GBM [82]. These studies support regional pharmacological access and early translational feasibility. They do not demonstrate a survival benefit attributable to ultrasound alone. Cross-study reporting should include the microbubble formulation and dose, acoustic frequency and pulse structure, power or pressure threshold, exposure duration, target volume, and timing of drug administration and imaging. The focal paracellular and transcellular permeability changes induced by FUS/MB are illustrated in Figure 2b.

Photodynamic modulation is another experimental means of increasing local barrier permeability. In non-tumor-bearing rats, 5-aminolevulinic acid-mediated photodynamic treatment produced a localized, dose-dependent BBB opening; experiments in healthy mice subsequently demonstrated leakage of albumin-bound dye and 70-kDa dextran [83,84]. These findings provide proof of barrier modulation, but they do not demonstrate delivery of a functional 150-kDa monoclonal antibody into GBM. The irradiation conditions, depth of treatment, duration of permeability, and risk of vascular or parenchymal injury require further assessment. For antibody delivery, this approach remains less clinically established than FUS/MB.

Photodynamic barrier opening should be distinguished from near-infrared photoimmunotherapy using an antibody–photosensitizer conjugate, which localizes a light-activated cytotoxic agent to antigen-positive cells. In an orthotopic mouse model derived from human glioblastoma stem cells, an AC133/CD133-targeting antibody–IR700 conjugate reached tumor tissue, and near-infrared illumination prolonged animal survival [85]. This was preclinical evidence of antigen-directed, light-dependent tumor killing, rather than evidence that photodynamic treatment opened the BBB or generated a therapeutic immune response in patients.

Nanocarriers may also transport therapeutic antibodies without changing the antibody’s molecular shuttle format. Polymeric nano-immunoconjugates carrying anti-PD-1 or anti-CTLA-4 antibodies improved survival relative to free antibodies in GL261 glioma-bearing mice [86]. However, the article is currently subject to an Editor’s Note questioning the reliability of the reported data; these findings should therefore be interpreted cautiously. A separate preclinical study reported increased brain delivery of exosome-associated bevacizumab and prolonged survival in animal glioma models [87]. Both examples involve antibody cargo, rather than nanoparticles that merely use an antibody as a targeting ligand. Their clinical value will depend on reproducible antibody loading and activity, regional tumor distribution, and safety after systemic administration.

As shown in Figure 2a,b, RMT and FUS/MB are complementary rather than interchangeable. RMT may support repeated systemic transport, but it introduces receptor- and construct-specific pharmacology. FUS/MB preserves the payload, but its effects depend on the acoustic dose, device workflow, and coverage of infiltrative tissue. Before larger efficacy trials, both strategies require paired spatial pharmacokinetic and pharmacodynamic measurements. These measurements should include drug concentration, target occupancy, and pathway modulation in enhancing and non-enhancing compartments. Such data are needed to distinguish delivery failure from target failure. After regional exposure has been demonstrated, the remaining question is whether the chosen antigen is sufficiently specific, accessible, and stable to produce a therapeutic effect.

### 3.2. Multi-Omic and Causal Approaches to Therapeutic Target Prioritization

Target discovery for antibody therapy should begin with the intended mechanism. A blocking antibody needs an accessible extracellular epitope and a pathway whose modulation has a relevant effect. An antibody–drug conjugate (ADC) additionally requires sufficient antigen density, uptake when intracellular payload release is intended, and susceptibility to its payload. Immune-modulating antibodies require a suitable immune-cell population and a favorable direction of modulation. Across formats, target coverage in tumor regions and treatment states must be weighed against expression in normal tissues. An association with GBM, even when reproducible across datasets, does not establish these properties [88,89].

Single-cell profiling helps identify the malignant states and host-cell populations in which a candidate is expressed. For example, CD24 was identified as a marker associated with a neural progenitor cell-like malignant state, but this observation did not test CD24-directed antibody treatment in GBM [90]. Longitudinal and spatial analyses add information about treatment-associated states, anatomical niches, and interactions between malignant and host cells [91,92]. A recent human spatial atlas, which included GBM as well as other CNS diagnoses, combined transcriptomic mapping with a prespecified surface-protein panel measured by CITE-seq. Such measurements strengthen evidence of protein expression in defined cell populations, but they do not establish therapeutic antibody activity [93]. Spatial interaction mapping has also implicated the ANXA1–FPR1 pathway near the invasive tumor zone. FPR1 protein was assessed in human GBM samples, and an FPR1-blocking antibody was tested in cultured human GBM isolates. The reported mouse survival effect, however, followed tumor-cell Fpr1 genetic inactivation rather than antibody administration [94].

Protein-level analysis provides a more direct test of accessibility and uptake than RNA abundance alone. Surfaceome and endocytome profiling of patient-derived GBM cultures identified CD44 as an abundant, internalizing surface protein and highlighted CXADR and CD47 in hypoxia-associated programs. The investigators also stained human tumor specimens and tested antibody uptake or antibody-directed payload delivery in cultured cells. For the cytotoxicity experiments, the MMAF payload was attached to an anti-IgG secondary antibody paired with a target-binding primary antibody. These experiments support antibody-relevant proof of concept in vitro; they do not establish the regional delivery, safety, or efficacy of an optimized ADC in animals or patients [88]. Accordingly, Table 2 includes only cell-surface candidates for which a target-binding antibody was used in a functional GBM experiment. Human-tissue expression, cultured-cell activity, and animal genetic evidence are distinguished rather than treated as equivalent validation.

Candidates without a functional antibody experiment in a GBM system remain valuable for biological prioritization, but are discussed in the text rather than included in Table 2. TREM2 analyses of glioma cohorts and myeloid cells support a potential role in the immune environment, without testing a therapeutic anti-TREM2 antibody in GBM [95]. PDCD1LG2, which encodes PD-L2, was identified within myeloid expression programs in a study that included both IDH-wildtype and IDH-mutant gliomas; this was not a test of PD-L2-directed therapy [96]. Brain-based Mendelian randomization (MR), transcriptome-wide association studies (TWAS), and proteome-wide association studies (PWAS) can further prioritize genes associated with glioma susceptibility [97,98,99]. Recent integrated tissue proteomic and metabolomic profiling paired with plasma metabolomics likewise identified glioma-associated biological programs and candidate biomarkers, but did not establish a validated cell-surface antibody target [100]. Even a statistically well-supported risk-associated gene remains a biological candidate until its relevant protein, extracellular accessibility, direction of antibody modulation, and activity against established GBM have been demonstrated.

A candidate should therefore advance to antibody-format development only after orthogonal tests establish the required surface or soluble protein expression, functional dependence, coverage across patients and tumor regions, and an acceptable normal-tissue profile. For ADCs designed for intracellular payload release, internalization and payload sensitivity should be assessed, whereas immune-dependent constructs require the relevant effector cells and functional context. Section 3.3 considers how these requirements can be tested experimentally and then linked to regional human exposure and safety.

### 3.3. Experimental and Translational Validation of Computationally Nominated Targets

A computationally nominated target becomes credible only when correlation is supported by causal and developability evidence. Validation should proceed through orthogonal perturbation, demonstration of antibody accessibility, and confirmation of target-dependent function. It should then advance to model-matched assessments of efficacy, exposure, and safety. Negative findings should be used to revise or redirect the hypothesis. HRH1 [101], PTP4A2 [102], and surfaceome or endocytome programs [88] illustrate complementary entry points. None alone establishes a valid therapeutic target.

HRH1 illustrates orthogonal validation. After nomination by WGCNA and receptor triage [101], two shRNAs suppressed proliferation, migration, invasion, and sphere formation. In an intracranial mouse model, HRH1 silencing reduced tumor burden and prolonged survival [101]. Terfenadine reproduced the phenotype, and histamine partly rescued it [101]. These findings reduced the likelihood of an shRNA-specific effect. Microenvironmental targets require lineage-resolved testing. CCL1 was assigned mainly to tumor-associated macrophages and AMFR to tumor cells. Receptor-discrimination experiments and lineage-specific deletion then linked this axis to tumor growth and immunosuppression [103].

Antibody tractability introduces a modality-specific requirement. HRH1 antibodies were screened for affinity and growth inhibition. The lead clone showed target-specific binding, as antibody-detection signal was lost in HRH1-knockout cells; it also localized to intracranial tumors and prolonged xenograft survival after intravenous administration [101]. These findings do not establish uniform exposure in humans, cross-reactivity, chronic toxicology, or immunogenicity. Human tumor xenografts in mice can assess tumor engagement, whereas immune-dependent mechanisms also require immunocompetent models.

Negative results can redirect development. PTP4A2 knockout and JMS-053 suppressed recurrent GBM growth in vitro, but systemic JMS-053 showed modest efficacy and poor BBB penetration [102]. PTP4A2 was a pathway-level finding rather than a directly accessible extracellular antibody target. Investigators therefore shifted along the PTP4A2–ROBO1 pathway to the surface-accessible target ROBO1. Knockout confirmed target dependence, and intracranial ROBO1 chimeric antigen receptor (CAR) T cells prolonged survival in an orthotopic mouse model [102]. These CAR T-cell findings support pathway-guided redesign but do not establish the efficacy of a ROBO1-directed antibody or clinical benefit. CCL1 neutralization also prolonged survival and reduced tumor-associated macrophage infiltration and FAK/PI3K-AKT signaling in immunocompetent GL261 mouse glioma and patient-derived xenograft mouse models [103]. Neither model replaces systemic toxicology.

Animal efficacy is not the final requirement. Human phase 0 tissue pharmacology can determine whether a systemically administered construct delivers active payload to the tumor. For example, intratumoral SN-38 was detected after sacituzumab govitecan, but the small nonrandomized study did not establish spatially uniform exposure or a survival benefit [29]. Translation should therefore link causal validation and antibody accessibility to model-matched efficacy, human exposure, safety, and redesign after failure. These findings should guide progression to larger efficacy trials, with construct redesign when exposure, target modulation, or safety is inadequate.

## 4. Future Perspectives: Emerging Antibody Formats and Translational Design Requirements

**Figure 3:**
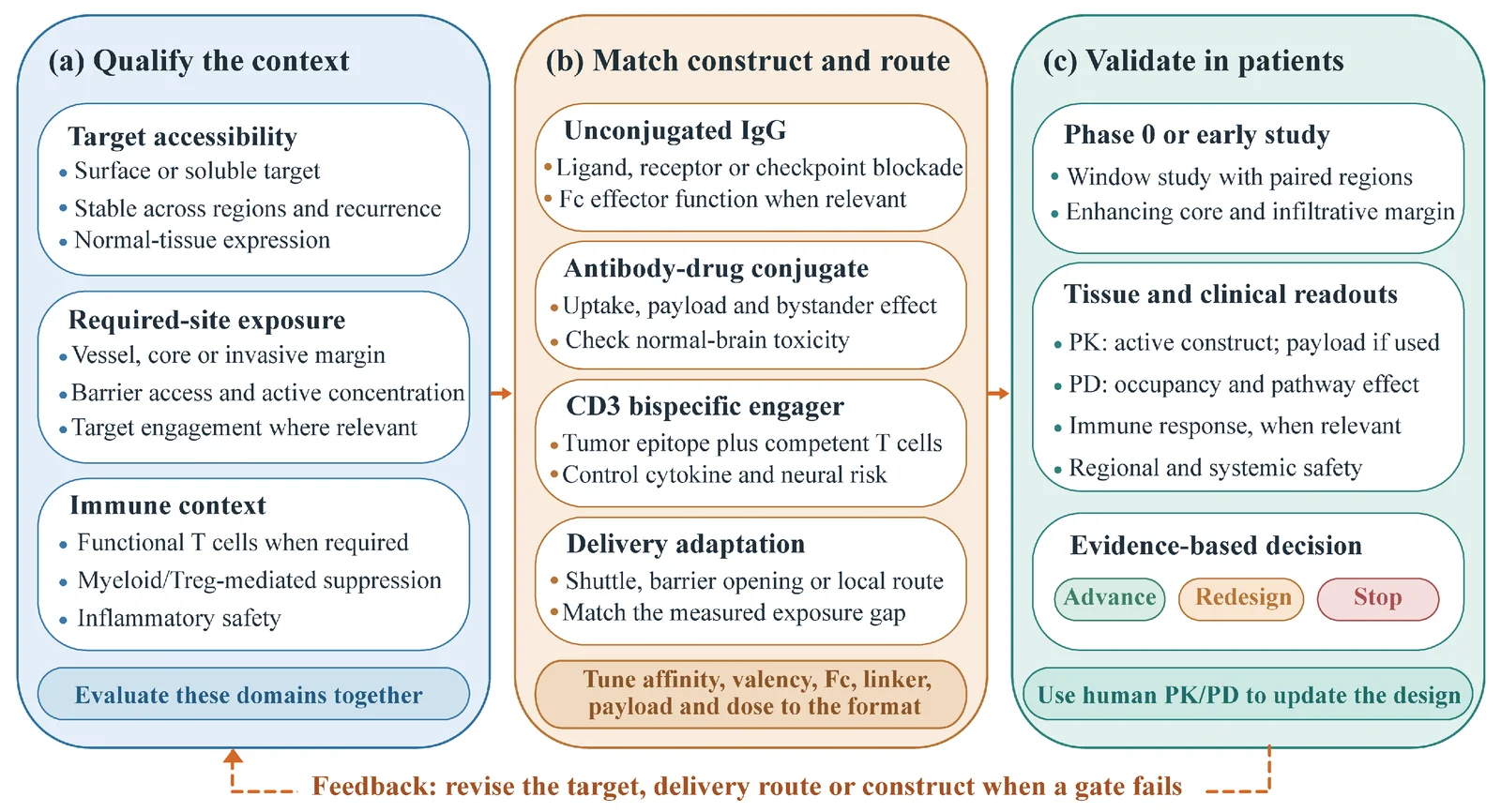
Proposed translational workflow for antibody therapeutics in glioblastoma. (**a**) Candidate qualification integrates extracellular target accessibility, spatial and longitudinal stability, exposure at the required site of action, and immune context when relevant. (**b**) These findings guide selection of an unconjugated immunoglobulin G (IgG), antibody–drug conjugate (ADC), CD3-engaging bispecific antibody, and delivery adaptation. (**c**) Phase 0 or early clinical studies assess regional drug exposure, target occupancy, downstream pharmacodynamics, immune effects when relevant, and safety, ideally in paired enhancing and infiltrative tissue. Results inform advance, redesign, or stop decisions. The diagram is a proposed framework rather than a validated clinical algorithm. Abbreviations: ADC, antibody–drug conjugate; CD3, cluster of differentiation 3; Fc, fragment crystallizable; IgG, immunoglobulin G; PD, pharmacodynamics; PK, pharmacokinetics; Treg, regulatory T cell.

Further progress requires iterative co-development of target selection, delivery strategy, and therapeutic format. Antigen selection, molecular architecture, effector mechanism, intratumoral pharmacology, and safety must be optimized together. Figure 3 summarizes an iterative translational workflow that links target accessibility and stability, regional exposure, immune context, construct design, and phase 0 or early clinical validation.

### 4.1. Antibody–Drug Conjugates: Target Selection, Payload Design and Intratumoral Exposure

ADCs can increase monoclonal-antibody cytotoxicity by coupling tumor recognition to a potent payload. However, their precision is conditional. Conventional intracellular-release ADCs generally require a tumor-selective, surface-accessible, sufficiently abundant, internalizing target that remains stable during treatment. Antibody affinity, drug-to-antibody ratio, linker stability, and payload susceptibility must also preserve a therapeutic window in normal brain tissue [28]. Target positivity alone is insufficient. Development should integrate spatial antigen density, internalization, and intratumoral exposure.

Orthotopic mouse studies of EGFR- and GPC1-directed MMAE ADCs support target-dependent activity. However, they remain preclinical demonstrations of construct feasibility [104,105]. Payload permeability creates a trade-off. A bystander effect may eliminate antigen-low cells, but an MMAE-containing EGFR ADC did not outperform a less diffusible comparator and caused greater toxicity in normal brain tissue. These findings were consistent with premature payload release in neural tissue [106]. Linker–payload combinations should therefore be evaluated in brain-relevant matrices rather than selected only for plasma stability or maximal cytotoxicity.

Human exposure is a decisive requirement. Sacituzumab govitecan delivered measurable SN-38 to resected recurrent GBM, but the study did not establish uniform exposure in infiltrative regions or a survival benefit [29]. Depatux-M also failed to improve overall survival in a phase III trial of EGFR-amplified disease. This result shows that antigen enrichment and progression-free or radiographic signals cannot replace confirmatory clinical benefit [16,107]. Convection-enhanced delivery and BTB modulation may increase exposure [108,109], but they cannot replace construct optimization. Neurotoxicity and a narrow therapeutic window also remain major limitations [106,108]. Trials should quantify conjugated antibody, free payload, target modulation, and regional pharmacodynamics together with safety and overall survival. Whereas ADCs add a cytotoxic payload, bispecific antibodies add a second recognition or effector function.

### 4.2. Bispecific Antibodies: Immune Redirection, Multi-Antigen Targeting and Brain Delivery

Bispecific antibodies provide a complementary strategy by combining two functions in one molecular system. Tumor-antigen × CD3 T-cell engagers can form an immune synapse and redirect cytotoxicity without requiring classical antigen presentation by tumor major histocompatibility complex class I (MHC-I). EGFRvIII × CD3 and IL-13Rα2 × CD3 constructs have shown dual-engagement-dependent killing in patient-derived GBM cell cultures and orthotopic mouse models. However, these findings remain preclinical. Their translation depends on target selectivity, access to functional effector cells, and control of inflammatory activation [110,111,112].

Antigen heterogeneity, molecular format, and brain exposure must be engineered together. Two parallel engagers directed against EGFRvIII × CD3 and HER2 × CD3 reduced single-antigen escape in preclinical models. A separate IL-13Rα2 × EGFRvIII × CD3 trispecific molecule achieved a similar goal through a single construct. These approaches represent distinct development strategies rather than interchangeable formats [113,114]. Systemically administered T-cell engagers have shown tumor-associated accumulation or extravascular retention in GBM models. These findings support BTB-associated delivery but do not establish universal passage across an intact BBB [110,115]. A monovalent, noncompetitive TfR × VEGF-Trap shows how a delivery arm and a therapeutic arm can be integrated. However, its safe extension to CD3 engagement remains unproven [116,117]. Local production by tumor-tropic neural stem cells provides another strategy to reduce systemic exposure and offset rapid clearance [112].

Clinical translation remains the decisive test. In the first-in-human EGFRvIII × CD3 study, the tested doses were tolerated, the maximum tolerated dose was not reached, and measurable cerebrospinal fluid exposure was observed. However, maximum serum concentrations remained below the predicted therapeutic range, and no radiographic responses were observed at the tested doses [118]. Some patients had sustained stable disease, but the phase I design and exposure below the predicted therapeutic range preclude firm conclusions about efficacy. Future constructs should jointly optimize tumor-arm valency, CD3 affinity, domain geometry, linker and Fc design, half-life, and dosing schedule. Trials should also prospectively monitor cytokine release syndrome, immune effector cell-associated neurotoxicity syndrome, seizures, and cerebral edema [117,118]. Exposure studies should distinguish among serum, cerebrospinal fluid, and spatially resolved pharmacologically active intratumoral concentrations [115,118].

Table 3 compares the required site of exposure, target stability, immune dependence, internalization requirements, and major safety risks across antibody modalities.

## 5. Conclusions and Perspectives

Monoclonal antibody therapy in GBM is unlikely to succeed through isolated optimization of delivery, target selection, or molecular format. Successful translation requires exposure at the site of action appropriate to each antibody modality. Tumor-directed constructs require evaluation of coverage across enhancing and infiltrative disease, whereas antiangiogenic and immune-modulating antibodies require assessments matched to their vascular or immune mechanisms. Target stability, effector competence, and construct-specific safety should be evaluated according to the intended mechanism. Where intratumoral target engagement is required, treatment-state-matched biomarkers and spatially resolved pharmacokinetic and pharmacodynamic measurements should assess active intratumoral exposure and target modulation. Phase 0 and early-phase trials should connect mechanism-relevant pharmacology and safety with a credible path to patient benefit. Radiographic and progression-free survival signals should be interpreted together with safety and overall survival.

## Figures and Tables

**Table 1:** Selected clinical trials and human evidence illustrating major barriers to antibody-based therapy in glioblastoma.

Major Barrier	Therapy/Target	Study Design/Population	Main Outcome	Core Interpretation	Ref.
**BBB/BTB** **delivery**	EMD55900 (MAb 425)/EGFR	Phase I; malignant brain tumors (*n* = 30)	0.0001–0.00001% ID/g	Target binding, but negligible tumor uptake.	[23]
	Cetuximab-IRDye800/EGFR	First-in-human imaging study; three brain tumors (two contrast-enhancing GBMs and one non-enhancing grade II diffuse astrocytoma)	Fluorescence detected in the enhancing GBMs but not in the non-enhancing astrocytoma	This comparison involved different tumor diagnoses and does not establish antibody exposure at the invasive margin of GBM; the study assessed imaging, not therapeutic efficacy.	[24]
	Bevacizumab/VEGF-A	RTOG 0825; randomized phase III; nGBM (*n* = 637)	Numerically longer PFS; prespecified significance threshold not met; no OS benefit.	The PFS difference did not meet the prespecified significance threshold, and no OS benefit was demonstrated.	[11]
	Depatuxizumab mafodotin/EGFR	INTELLANCE-1; randomized phase III; EGFR-amplified nGBM (*n* = 639)	PFS signal; no OS gain	Preclinical data support heterogeneous intracranial exposure as a possible contributor to reduced efficacy.	[2,16]
	Serclutamab talirine/EGFR	Phase I; advanced GBM *(n* = 24)	1 PR; minimal activity	Intratumoral exposure was not established.	[43]
	AMG 595/EGFRvIII	First-in-human phase I; EGFRvIII-positive rGBM (*n* = 32)	2 PR; 15 SD	Whole-tumor payload coverage remained uncertain.	[44]
	Losatuxizumab vedotin/EGFR	Phase I; EGFR-expressing solid tumors; GBM subset (*n* = 5)	Trial stopped early because of infusion reactions; GBM-specific outcome not reported.	The GBM subset was too small and insufficiently reported to assess activity.	[45]
**Spatiotemporal** **heterogeneity**	Multiregional biopsy/EGFR-PDGFRA-PTEN	Human spatial-mapping study; GBM (74 patients; 318 biopsies)	Regional target discordance	Regional discordance suggests that one biopsy may not represent whole-tumor target status.	[32]
	Paired primary-recurrent profiling/EGFRvIII	Paired primary–recurrent cohort; GBM (42 EGFRvIII-evaluable pairs)	EGFRvIII lost in ~50%	EGFRvIII expression was not retained in about half of initially positive tumors at recurrence.	[3]
	Rindopepimut */EGFRvIII	ACT IV; randomized phase III; EGFRvIII-positive nGBM (*n* = 745)	No survival gain	EGFRvIII loss was similar between groups and was not associated with outcome.	[39]
	Depatuxizumab mafodotin/EGFR	M12-356 Arm C; phase I; EGFR-amplified rGBM (*n* = 66)	ORR 6.8%; PFS6 28.8%	EGFRvIII status did not predict response in this cohort.	[40]
	Depatuxizumab mafodotin + TMZ/EGFR	M12-356; phase I; EGFR-amplified rGBM (*n* = 60)	ORR ~14%; PFS6 25.2%	Predictive value of baseline EGFR biomarkers was not established in this phase I study.	[41]
	Depatuxizumab mafodotin/EGFR	INTELLANCE-1; randomized phase III; EGFR-amplified nGBM (*n* = 639)	PFS signal; no OS gain	The trial did not assess clonal repopulation as an explanation for the lack of OS benefit.	[16]
	Nivolumab/PD-1	CA209-9UP; window-of-opportunity phase II; recurrent high-grade glioma (*n* = 8 patients; 16 paired samples)	Mesenchymal/immune shifts	Nivolumab was associated with mesenchymal and immune-state shifts; direct selection was not established.	[42]
**Immunosuppressive** **TME**	Nivolumab/PD-1	CheckMate 498; randomized phase III; MGMT-unmethylated nGBM (*n* = 560)	OS 13.4 vs. 14.9 months	PD-1 blockade could not replace TMZ; PD-L1 expression did not identify a benefiting subgroup in this trial.	[14]
	Pembrolizumab +/− bevacizumab/PD-1, VEGF-A	Randomized phase II; bevacizumab-naïve rGBM (*n* = 80)	PFS6 26.0% vs. 6.7%; no OS tail	Pembrolizumab showed limited activity with or without bevacizumab; redundant suppression was not directly tested.	[46]
	Bavituximab/phosphatidylserine	Single-arm phase II; IDH-wildtype nGBM (*n* = 33 evaluable)	MDSCs decreased; mOS 15.4 months	Myeloid modulation occurred, but clinical benefit was unproven.	[47]

Note: Barriers are non-exclusive; negative or limited activity does not prove a single cause. Studies using historical GBM diagnoses or mixed malignant brain tumor and high-grade glioma populations are described as originally reported; not all participants necessarily met the current WHO definition of IDH-wildtype GBM. * Rindopepimut is a peptide vaccine included as a clinical analogue of single-antigen escape. Abbreviations: BBB, blood–brain barrier; BTB, blood–tumor barrier; EGFR, epidermal growth factor receptor; EGFRvIII, epidermal growth factor receptor variant III; GBM, glioblastoma; ID/g, injected dose per gram; IDH, isocitrate dehydrogenase; MDSC, myeloid-derived suppressor cell; MGMT, O6-methylguanine-DNA methyltransferase; mOS, median overall survival; nGBM/rGBM, newly diagnosed/recurrent GBM; ORR, objective response rate; OS, overall survival; PD-1, programmed cell death protein 1; PD-L1, programmed death ligand 1; PFS, progression-free survival; PFS6, 6-month PFS; PR/SD, partial response/stable disease; RTOG, Radiation Therapy Oncology Group; TME, tumor microenvironment; TMZ, temozolomide; VEGF-A, vascular endothelial growth factor A.

**Table 2:** Selected GBM cell-surface candidates with direct functional antibody testing in experimental systems.

Discovery Method	Candidate/Compartment	Surface or Tissue Evidence	GBM Antibody Experiment	Main Remaining Gap	Ref.
**Spatial interaction mapping**	FPR1/invasive-margin GBM cells	FPR1 protein in human GBM; receptor blockade in culture	Neutralizing anti-FPR1 in primary human GBM cultures	In vivo antibody delivery, efficacy, and safety	[94]
**Surfaceome/endocytome mapping**	CD44/malignant cells	Surface abundance and antibody uptake in patient-derived cultures	Antibody-guided MMAF delivery; variable killing in vitro	Patient coverage, in vivo delivery, and safety	[88]
**Hypoxic surfaceome**	CXADR/hypoxic GBM cells	Surface protein in patient-derived cultures; human GBM staining	Antibody-guided MMAF delivery; greater killing under hypoxia in vitro	Spatial coverage, in vivo efficacy, and safety.	[88]
**Hypoxic surfaceome**	CD47/hypoxic GBM cells	Hypoxia-associated surface protein; human GBM staining	Antibody-guided MMAF delivery; greater killing under hypoxia in vitro	Normal-tissue selectivity, in vivo efficacy, and safety	[88]

Note: CD44, CXADR, and CD47 were tested in cultured GBM cells using target-binding primary antibodies paired with an MMAF-linked secondary antibody. These were surrogate ADC assays, not tests of optimized therapeutic ADCs. Anti-FPR1 was tested in human GBM cultures; the reported mouse survival effect followed genetic Fpr1 inactivation, not antibody treatment. None of these studies establishes clinical antibody efficacy in GBM. Abbreviations: ADC, antibody–drug conjugate; GBM, glioblastoma; MMAF, monomethyl auristatin F.

**Table 3:** Format-specific delivery, target, effector, internalization, and safety requirements of antibody-based strategies in glioblastoma.

Format/Example	Required Exposure	Target Stability	Immune Reliance	Internalization	Main Safety Risk	Ref.
**Anti-VEGF-A IgG (bevacizumab)**	Vascular/extracellular VEGF-A; uniform brain entry is unnecessary for vascular action.	Tumor-antigen loss is less relevant; alternative vascular or invasive programs may persist.	Low for VEGF-A blockade.	No.	Hypertension, vascular events and impaired wound healing.	[10,11]
**Anti-PD-1 IgG (nivolumab)**	Relevant immune sites; functional T cells must reach the tumor.	PD-1 availability and effector T-cell state matter; tumor PD-L1 is a variable contextual biomarker, not the antibody target.	High; competent T cells are essential.	No for blockade.	Immune adverse events; possible neuroinflammation.	[14,46]
**Tumor-directed IgG (anti-EGFR)**	Antigen-positive cells across core and invasive margin.	High; epitope heterogeneity and loss limit coverage.	Variable; Fc-mediated killing needs effectors.	Mechanism dependent.	Normal-tissue effects; safety is construct-specific.	[8,23,24]
**ADC (Depatux-M; sacituzumab govitecan)**	Active conjugate or payload across tumor regions.	High; bystander killing may partly offset antigen heterogeneity.	Lower for direct payload killing.	Usually yes for intracellular-release designs.	Payload toxicity, including ocular and possible neural injury.	[2,16,28,29,106]
**CD3 bispecific (EGFRvIII × CD3)**	Construct, tumor epitope and functional T cells must colocalize.	High; multi-antigen targeting may reduce escape.	Essential.	No for immune synapse.	CRS, neurotoxicity, edema or seizures, and off-tumor activation.	[113,117,118]

Note: Entries summarize construct-dependent requirements, not proven causes of trial failure. Anti-EGFR IgG studies assess access or imaging; the EGFRvIII × CD3 trial is early-phase. BBB shuttles and rindopepimut (a peptide vaccine) are excluded. Abbreviations: ADC, antibody–drug conjugate; CRS, cytokine release syndrome; Depatux-M, depatuxizumab mafodotin; IgG, immunoglobulin G.

## Data Availability

No new data were created or analyzed in this study. Data sharing is not applicable to this article.

## Abbreviations

The following abbreviations are used in this manuscript:

ADC	Antibody–drug conjugate
BBB	Blood–brain barrier
BTB	Blood–tumor barrier
CAR	Chimeric antigen receptor
CRISPR	Clustered regularly interspaced short palindromic repeats
EGFR	Epidermal growth factor receptor
EGFRvIII	Epidermal growth factor receptor variant III
Fc	Fragment crystallizable
FcRn	Neonatal Fc receptor
FUS	Focused ultrasound
GBM	Glioblastoma
HER2	Human epidermal growth factor receptor 2
ID/g	Injected dose per gram
IDH	Isocitrate dehydrogenase
IgG	Immunoglobulin G
IHC	Immunohistochemistry
mAb	Monoclonal antibody
MDSC	Myeloid-derived suppressor cell
MES	Mesenchymal
MGMT	O6-methylguanine-DNA methyltransferase
MHC-I	Major histocompatibility complex class I
nGBM	Newly diagnosed glioblastoma
ORR	Objective response rate
OS	Overall survival
PD-1	Programmed cell death protein 1
PD-L1	Programmed death ligand 1
PD-L2	Programmed death ligand 2
PET	Positron emission tomography
PFS	Progression-free survival
PFS6	Progression-free survival at 6 months
PR	Partial response
rGBM	Recurrent glioblastoma
RMT	Receptor-mediated transcytosis
SD	Stable disease
TAM	Tumor-associated macrophage
TME	Tumor microenvironment
TMZ	Temozolomide
VEGF	Vascular endothelial growth factor
